# Lethal Injection: Other Views

**DOI:** 10.1371/journal.pmed.0040224

**Published:** 2007-06-26

**Authors:** The PLoS Medicine Editors

The recently published research article on lethal injection [1] and our editorial commentary [2] both produced a number of short letters via our electronic reader response system.

There were five letters commenting on the research article. All were written by strong supporters of the death penalty who took the view that, as the victims of those who had been convicted of murder had suffered, the perpetrators should themselves experience pain as well as execution. We posted two of these letters on our Web site, but felt the other three were written in terms that made them unsuitable for inclusion.

Our editorial attracted 11 letters. Three supported the views we had expressed, one commented on a small factual error, and seven were hostile, again focusing on the desirability of making murderers suffer. We felt that one of the supportive letters (above) was of particular interest and we have therefore chosen to highlight it by means of formal publication in the Correspondence section of the journal. Of the seven hostile letters, we considered that four were suitable for posting on the Web site.

We do not intend, within our Correspondence section, to publish further letters commenting on the research article or the editorial. However, as with all the articles we publish, reader responses for the Web site may be submitted at any time. After a very brief screening for suitability, reader responses appear on the site within a day or two of submission.
